# Paradoxes in dynamic stability of mechanical systems: investigating the causes and detecting the nonlinear behaviors

**DOI:** 10.1186/s40064-016-1684-9

**Published:** 2016-01-21

**Authors:** Angelo Luongo, Manuel Ferretti, Francesco D’Annibale

**Affiliations:** International Research Center on Mathematics and Mechanics of Complex Systems, Via Giovanni Gronchi, 18, L’Aquila, Italy

**Keywords:** Ziegler paradox, Nicolai paradox, Piezoelectric control, Eigenvalue sensitivity, Semi-simple eigenvalues, Defective eigenvalues, Hopf bifurcation, Post-critical behavior

## Abstract

A critical review of three paradoxical phenomena, occurring in the dynamic stability of finite-dimensional autonomous mechanical systems, is carried out. In particular, the well-known destabilization paradoxes of Ziegler, due to damping, and Nicolai, due to follower torque, and the less well known failure of the so-called ‘principle of similarity’, as a control strategy in piezo-electro-mechanical systems, are discussed. Some examples concerning the uncontrolled and controlled Ziegler column and the Nicolai beam are discussed, both in linear and nonlinear regimes. The paper aims to discuss in depth the reasons of paradoxes in the linear behavior, sometimes by looking at these problems in a new perspective with respect to the existing literature. Moreover, it represents a first attempt to investigate also the post-critical regime.

## Background

There exist several paradoxes in mechanics; the most amazing may occur in stability analysis. A celebrated problem is the ‘Ziegler Paradox’ (Ziegler 1952; Beck 1952; Bolotin 1963; Herrmann and Jong 1965; Herrmann 1967; Leipholz 1964; Plaut and Infante 1970; Plaut 1971; Walker 1973; Hagedorn 1970; Banichuk et al. 1989; Kounadis 1992), also known as the ‘destabilizing effect of damping’ , according to which a given small damping has a detrimental effect on the stability of linear circulatory systems. The Ziegler column, i.e. an upward double pendulum loaded at the tip by a follower force, represents a discrete mechanical prototype of this paradox. A geometrical explanation of the phenomenon, based on the existence of the ‘Whitney’s umbrella’ surface (Whitney 1943), was given by Bottema (1955, 1956). More recently, Seyranian and Kirillov, according to the singularity theory by Arnold (1983, 1992), gave a justification of the paradox for both general finite-dimensional (Seyranian and Mailybaev 2003; Kirillov 2005, 2013; Kirillov and Seyranian 2005) and continuous (Kirillov and Seyranian 2005) systems, based on a perturbation analysis of the eigenvalues at the (known) double Hopf bifurcation point of the circulatory system. Differently from this latter approach, a perturbation algorithm was developed in Luongo and D’Annibale (2014, 2015) and Andreichikov and Yudovich (1974), where the starting point of the asymptotic analysis is an unknown, marginally stable, sub-critical undamped system.

A less known, but equally surprising phenomenon, is the ‘Nicolai Paradox’ (Nicolai 1928, 1929; Bolotin 1963). It concerns a cantilever beam, of equal moments of inertia, loaded at the tip by a follower torque, which causes dynamic instability of the trivial equilibrium at a critical value equal to zero. The amazing phenomenon has been recently reconsidered by Seyranian and Mailybaev (2011), who, according to the singularity theory Arnold (1983), proved that this paradox is related to the bifurcation of a double semi-simple eigenvalue, leading to a stability domain with a conic singularity. Moreover, the effects of the pre-twist deformation, the damping, an axial dead load and the compressibility of the beam have been deeply analyzed in Seyranian et al. (2014), Luongo et al. (2014) and Seyranian and Glavardanov (2014). An extension of the problem to second-order perturbations was also performed in Luongo and Ferretti (2014).

A third paradox, recently discovered by the authors of the present paper, concerns the stability of *autonomous* piezo-electro-mechanical (PEM) systems in the presence of nonconservative (positional) actions (D’Annibale et al. 2015). It has been proved that the so-called ‘similarity principle’ (see, e.g., Alessandroni et al. 2004, 2005; Andreaus et al. 2004; dell’Isola et al. 2003a, b, 2004; Maurini et al. 2004; Porfiri et al. 2004; Rosi and Pouget 2010; Alessandroni et al. 2002), which usually works in controlling vibrations of externally excited (i.e. *non autonomous*) systems, has instead detrimental effects on the occurrence of dynamic bifurcations. Said in other words, the connection of a similar piezo-electric system to a mechanical one, the former duplicating the whole spectrum of the eigenvalues of the latter, which would supply a complete protection from *any* excitation frequency, is indeed detrimental in terms of stability.

In spite of a wide literature existing on paradoxical linear systems, to the authors’ knowledge, really few studies concerning the post-critical analysis have been carried out (see, e.g., Hagedorn 1970; Thomsen 1995; O’Reilly et al. 1996). The main question to be answered is the following: does the paradoxical loss of stability predicted by the linear theory really lead to motions of large amplitude when nonlinearities are accounted for? In other terms, since the amplitudes of motions depend on nonlinearities, the question is to ascertain if nonlinearities are able or not to limit the amplitudes within values smaller than a certain tolerance. A comprehensive answer, of course, would require an in-depth study which is beyond the scope of this work; anyway, a first attempt in this direction is made here by limiting ourselves to a numerical investigation.

The scope of this paper is twofold. First, we want to frame our previous results on linear stability analysis of paradoxical systems, so far developed independently, in a unique organic context; second, we want to illustrate some original, although so far limited, results concerning nonlinear behavior. To these ends, the above mentioned phenomena are reviewed for a class of finite-dimensional mechanical systems. The reasons of the paradoxes are explained by recalling asymptotic expansions of the eigenvalues of the tangent operator previously developed in the literature. Preliminary results concerning the nonlinear behavior are obtained via numerical analyses, directly carried out on the equations of motion of prototype systems. The limit-cycle which arises after the occurrence of (simple or semi-simple) Hopf bifurcations, is determined, and the influence of the main parameters is studied. It is wished that the studies presented here will stimulate also experimental activity, necessary to validate the theoretical predictions.

The paper is organized as follows. In second section, the model of a class of finite-dimensional mechanical system, suffering the paradoxes discussed above, is introduced. In third and fourth sections, the Ziegler and Nicolai paradoxes are investigated, respectively. In fifth section, the failure of the ‘similarity principle’ is discussed. In sixth section, some conclusions are drawn. Finally an “Appendix” furnishes details.

## The model

### Uncontrolled systems

We consider a class of $n_{m}$-dimensional autonomous mechanical systems, whose nondimensional equations of motion are in the following form:

1 $$\begin{aligned} {\mathbf{M}}{{\ddot{\mathbf{x}}}}+{{\mathbf{C}}}{{\dot{\mathbf{x}}}}+\left( {{\mathbf{K}}}+\mu {{\mathbf{H}}}\right) {\mathbf{x}}={{\mathbf{F}}}_{1}\left( {\mathbf{x}},{\mathbf{x}},{\mathbf{x}}\right) +{\mathbf{F}}_{2}\left( {\mathbf{x}},{\dot{\mathbf{x}}},{\dot{\mathbf{x}}}\right) +{\mathbf{F}}_{3}\left( {\mathbf{x}},{\mathbf{x}},{\ddot{\mathbf{x}}}\right) \end{aligned}$$

where a dot denotes differentiation with respect the time $t; {\mathbf{x}}={\mathbf{x}}\left( t\right)$ is the $n_{m}\times 1$ column matrix of the mechanical Lagrangian coordinates; ${\mathbf{M}}, {{\mathbf{K}}}, {{\mathbf{C}}}$ are the $n_{m}\times n_{m}$ symmetric mass, stiffness and damping matrices, respectively; ${\mathbf{H}}$ is the $n_{m}\times n_{m}$ non-symmetric circulatory matrix; $0<\mu \in \mathbb {R}$ is the load parameter; ${\mathbf{F}}_{j}\left( \cdot \right) ,\, j=1,\ldots ,3$ are $n_{m}\times 1$ trilinear vector functions of their arguments, accounting for geometrical nonlinearities, viz. ${\mathbf{F}}_{1}$, and inertial forces, viz. ${\mathbf{F}}_{2},\, {\mathbf{F}}_{3}$.

**Fig. 1:**
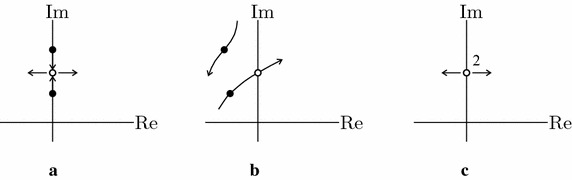
Sketch of the behavior of eigenvalues close to a bifurcation point: **a** circulatory Hopf of an undamped system; **b** simple Hopf of a damped system; **c** semi-simple Hopf

 The trivial equilibrium position ${\mathbf{x}}=\mathbf{0}$ of system (1) can lose stability according to one of the three mechanisms sketched in Fig. 1, as discussed ahead.

### Controlled systems

When the mechanical system (1) is connected, via a set of piezoelectric devices, to an active electrical circuit, a PEM system is obtained, whose linear part is of the type discussed, e.g., in D’Annibale et al. (2015). The equations of motion assume the following non-dimensional form [see also D’Annibale et al. (2015) for a detailed derivation]:

2 $$\begin{aligned} \begin{aligned}{\left\{ \begin{array}{ll} {\mathbf{M}}_{m}{\ddot{\mathbf{x}}}+{\mathbf{C}}_{m}{\dot{\mathbf{x}}}+\left( {\mathbf{K}}_{m}+\mu _{m}{\mathbf{H}}_{m}\right) {\mathbf{x}}-\gamma {{\mathbf{G}}}^{T}{{\dot{\mathbf{y}}}}={\mathbf{F}}_{1}\left( {\mathbf{x}},{\mathbf{x}},{\mathbf{x}}\right) +{\mathbf{F}}_{2}\left( {\mathbf{x}},{\dot{\mathbf{x}}},{\dot{\mathbf{x}}}\right) \\ +{\mathbf{F}}_{3}\left( {\mathbf{x}},{\mathbf{x}},{\ddot{\mathbf{x}}}\right) \\ {\mathbf{M}}_{e}{{\ddot{\mathbf{y}}}}+{\mathbf{C}}_{e}{{\dot{\mathbf{y}}}}+\left( {\mathbf{K}}_{e}+\mu _{e}{\mathbf{H}}_{e}\right) {\mathbf{y}}+\gamma {\mathbf{G}}{\dot{\mathbf{x}}}=\mathbf{0} \end{array}\right. }\end{aligned} \end{aligned}$$

Here, and in the following, the subscripts “*m*”, “*e*” refer to mechanical and electrical quantities, respectively. ${\mathbf{x}}\left( t\right) , {\mathbf{y}}\left( t\right)$ are the Lagrangian coordinates of the PEM system, namely: ${\mathbf{x}}\left( t\right)$ is the $n_{m}$-dimensional column-matrix of the Lagrangian coordinates of the structure and ${\mathbf{y}}\left( t\right)$ is the $n_{e}$-dimensional column-matrix of the flux linkages at the nodes of the circuit. Moreover, ${\mathbf{M}}_{\alpha }, {\mathbf{K}}_{\alpha }, {\mathbf{C}}_{\alpha } (\alpha =m,e)$ are the $n_{\alpha }\times n_{\alpha }$ mass, stiffness and damping matrices for mechanical and electrical sub-systems, respectively; ${\mathbf{H}}_{m}$ is the $n_{m}\times n_{m}$ circulatory mechanical matrix, accounting for external positional nonconservative forces, whose amplitude is governed by the load multiplier $\mu _{m}; {\mathbf{H}}_{e}$ is the $n_{e}\times n_{e}$ circulatory electrical matrix, accounting for the nonconservative actions furnished to the system by the active electrical circuit, whose amplitude is governed by the load multiplier $\mu _{e}; {\mathbf{G}}$ is a $n_{e}\times n_{m}$ electro-mechanical coupling matrix, here referred to as the ‘gyroscopic matrix’, whose amplitude depends on a (small) parameter $\gamma$. While ${\mathbf{M}}_{\alpha }, {\mathbf{K}}_{\alpha }$ and ${\mathbf{C}}_{\alpha }$ are symmetric matrices, ${\mathbf{H}}_{m}$ and ${\mathbf{H}}_{e}$ are not. ${\mathbf{G}}$, instead, is not squared, unless $n_{m}=n_{e}$; however, even in this case, it is generally non-symmetric. Finally the apex *T* denotes the transpose matrix.

By following the lines of D’Annibale et al. (2015), the similarity between mechanical (primary) and electrical (secondary) sub-systems is obtained *when the coefficients of the uncoupled linear parts of Eq. (*2*) are identical*. This circumstance occurs when $n_{m}=n_{e}$ and the following properties hold: (a) equal *own characteristics*, ${\mathbf{M}}_{m}={\mathbf{M}}_{e}=:{\mathbf{M}}, {\mathbf{K}}_{m}={\mathbf{K}}_{e}=:{\mathbf{K}}$; (b) equal *damping characteristics*, ${\mathbf{C}}_{m}={\mathbf{C}}_{e}=:{\mathbf{C}}$; (c) equal *external actions characteristics*, ${\mathbf{H}}_{m}={\mathbf{H}}_{e}=:\mathcal {{\mathbf{H}}}$, with $\mu :=\mu _{m}=\mu _{e}$. For these systems, Eq. (2) reduce to:

3 $$\begin{aligned} \begin{aligned}{\left\{ \begin{array}{ll} {\mathbf{M}}{\ddot{\mathbf{x}}}+{\mathbf{C}}{\dot{\mathbf{x}}}+\left( {\mathbf{K}}+\mu {\mathbf{H}}\right) {\mathbf{x}}-\gamma {\mathbf{G}}^{T}{\dot{\mathbf{y}}}={\mathbf{F}}_{1}\left( {\mathbf{x}},{\mathbf{x}},{\mathbf{x}}\right) +{\mathbf{F}}_{2}\left( {\mathbf{x}},{\dot{\mathbf{x}}},{\dot{\mathbf{x}}}\right) +{\mathbf{F}}_{3}\left( {\mathbf{x}},{\mathbf{x}},{\ddot{\mathbf{x}}}\right) \\ {\mathbf{M}}{{\ddot{\mathbf{y}}}}+{\mathbf{C}}{\dot{\mathbf{y}}}+\left( {\mathbf{K}}+\mu {\mathbf{H}}\right) {\mathbf{y}}+\gamma {\mathbf{G}}{\dot{\mathbf{x}}}=\mathbf{0} \end{array}\right. }\end{aligned} \end{aligned}$$

The trivial equilibrium position ${\mathbf{x}}={\mathbf{y}}=\mathbf{0}$ of system (3) can lose stability via the mechanism illustrated in Fig. 1c, as discussed ahead.

## The Ziegler paradox

This section is devoted to recall the well-known Ziegler paradox, with the aim to highlight some important aspects both in the linear and nonlinear regimes.

**Fig. 2:**
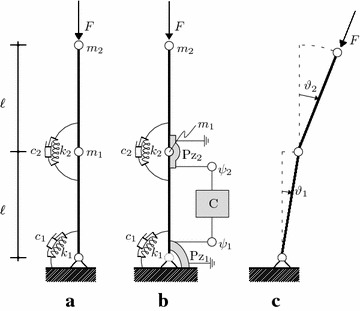
The Ziegler column: **a** uncontrolled column; **b** controlled column; **c** Lagrangian coordinates

The system that we will take as a prototype of such a paradox is the so called Ziegler column (Ziegler 1952), depicted in Fig. 2a. It consists of a two hinged weightless rigid bars of equal length $\ell$, carrying two concentrated masses, $m_{1}:=2m$ at the common hinge, and $m_{2}:=m$ at the tip; it is visco-elastically constrained at the hinges by: (a) two linear springs of stiffness $k_{1}:=k$ and $k_{2}:=k$ and (b) two linear dashpots of viscosity coefficients $c_{1}$ and $c_{2}$, respectively. Moreover, the column is loaded at the free end by a follower force of intensity *F*, whose direction remains parallel to the upper bar.

The equations of motion for the system are expressed in terms of its Lagrangian coordinates, namely the rotations of the two bars viz. $\vartheta _{1}$ and $\vartheta _{2}$, (see Fig. 2c). When an expansion up to the cubic terms in displacements and velocities of the exact kinematics is carried out, the equations of motion assume the form of Eq. (1), and the following definitions hold (see, e.g., Luongo and D’Annibale 2014; Hagedorn 1970):

4 $$\begin{aligned} \begin{aligned} {\mathbf{x}}&:=\left[ \begin{array}{c} \vartheta _{1}\\ \vartheta _{2} \end{array}\right] \\ {\mathbf{M}}&:=\left[ \begin{array}{cc} 3 &{} 1\\ 1 &{} 1 \end{array}\right] ,\quad {\mathbf{K}}:=\left[ \begin{array}{cc} 2 &{} -1\\ -1 &{} 1 \end{array}\right] \\ {\mathbf{C}}&:=\left[ \begin{array}{cc} \xi _{1}+\xi _{2} &{} -\xi _{2}\\ -\xi _{2} &{} \xi _{2} \end{array}\right] ,\quad {\mathbf{H}}:=\left[ \begin{array}{cc} -1 &{} 1\\ 0 &{} 0 \end{array}\right] \\ {\mathbf{F}}_{1}\left( {\mathbf{x}},{\mathbf{x}},{\mathbf{x}}\right)&:=\mu \left[ \begin{array}{c} -\frac{1}{6}\left( \vartheta _{1}-\vartheta _{2}\right) {}^{3}\\ 0 \end{array}\right] \\ {\mathbf{F}}_{2}\left( {\mathbf{x}},{\dot{\mathbf{x}}},{\dot{\mathbf{x}}}\right)&:=\left[ \begin{array}{c} -\left( \vartheta _{1}-\vartheta _{2}\right) {{\dot{\vartheta }}}_{2}^{2}\\ \left( \vartheta _{1}-\vartheta _{2}\right) {\dot{\vartheta }}_{1}^{2} \end{array}\right] \\ {\mathbf{F}}_{3}\left( {\mathbf{x}},{\mathbf{x}},{\ddot{\mathbf{x}}}\right)&:=\left[ \begin{array}{c} \frac{1}{2}\left( \vartheta _{1}-\vartheta _{2}\right) {}^{2}{\ddot{\vartheta }}_{2}\\ \frac{1}{2}\left( \vartheta _{1}-\vartheta _{2}\right) {}^{2}{\ddot{\vartheta }}_{1} \end{array}\right] \end{aligned} \end{aligned}$$

together with the quantities defined in the next, which have to be introduced for nondimensionalization:

5 $$\begin{aligned} \begin{aligned}&\begin{aligned}\tau =\omega t,\quad \omega ^{2}=\frac{k}{m\ell ^{2}},\end{aligned} \quad \mu =\frac{F}{m\ell \omega },\quad \xi _{1}=\frac{c_{1}}{m\ell ^{2}\omega },\quad \xi _{2}=\frac{c_{2}}{m\ell ^{2}\omega } \end{aligned} \end{aligned}$$

Here $\xi _{1}$ and $\xi _{2}$ are the two damping coefficients, thus entailing ${\mathbf{C}}={\mathbf{C}}\left( \xi _{1},\xi _{2}\right)$.

### Linear analysis

Let us first consider the linearized equations (1), with the aim to discuss the bifurcation mechanism occurring in the paradox. When the system is undamped (also referred to as circulatory), i.e. ${\mathbf{C}}=\mathbf{0}$, and $\mu =0$, the two pairs of complex conjugate eigenvalues lie on the imaginary axis so that the system is (marginally) stable. If $\mu$ is increased from zero, the eigenvalues move on the imaginary axis, still remaining distinct (see Fig. 1a), until the load reaches a critical value, namely $\mu =\mu _{c}$, at which they collide and a * circulatory (or reversible) Hopf bifurcation* takes place; if an infinitesimal increment $\delta \mu >0$ is given, they separate and instability occurs. The load value $\mu _{c}$ is the critical load of the circulatory system.

When the system is damped, namely ${\mathbf{C}}$ is positive definite, there exists a critical load $\mu _{d}$, that is the smallest $\mu$ at which an eigenvalue (together with its complex conjugate) crosses from the left the imaginary axis, (see Fig. 1b) and a* simple Hopf bifurcation* occurs. When the damping is sufficiently small, $\mu _{d}<\mu _{c}$, as it has been show in several contributions in the literature (see, e.g., Ziegler 1952; Bolotin 1963; Herrmann and Jong 1965; Seyranian and Mailybaev 2003; Kirillov and Verhulst 2010; Kirillov 2005).

**Fig. 3:**
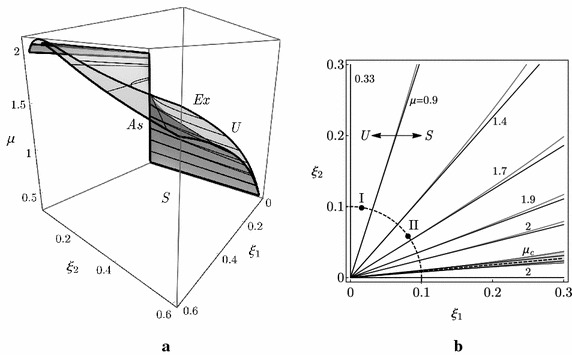
Stability boundary of the Ziegler column: **a** critical manifold in the $(\mu ,\xi _{1},\xi _{2})$-parameter space: *S* stable region, *U* unstable region, *Ex* exact manifold, *As* asymptotic manifold; **b** $\mu$-isolines: *S* stable region, *U* unstable region

The linear stability analysis of the column can be tackled through an exact or an asymptotic analysis. The exact analysis, carried out by making use of the Routh–Hurwitz criterion on the characteristic equation of the eigenvalue problem associated with the linearized Eq. (1) (see, e.g., Kirillov 2005), furnishes the critical locus in the $\left( \mu ,\xi _{1},\xi _{2}\right)$-space, known in the literature as the ‘Whitney’s umbrella’ surface (Whitney 1943; Kirillov and Verhulst 2010), whose equation reads:

6 $$\begin{aligned} \mu =\mu _{c}+\frac{{\xi _{1}}{\xi _{2}}}{2}-\frac{\left( 3-2\sqrt{2}\right) }{2}\frac{\left( {\xi _{1}}-\left( 4+5\sqrt{2}\right) {\xi _{2}}\right) ^{2}}{({\xi _{1}}+{\xi _{2}})({\xi _{1}}+6{\xi _{2}})} \end{aligned}$$

where $\mu _{c}:=7/2-\sqrt{2}\simeq 2.09$. The locus is displayed in Fig. 3. In particular, Fig. 3a shows the exact Whitney’s umbrella surface (labeled by *Ex*), given by Eq. (6), in the 3D domain $\left( \mu ,\xi _{1},\xi _{2}\right)$; it separates the stable region (marked with *S* in the figure) from the unstable one (marked with *U* in the figure). The points on the surface are Hopf bifurcation points, except for those on the $\mu$-axis that, indeed, are marginally-stable points, for which a circulatory Hopf bifurcation occurs at $\mu =\mu _{c}$ (Luongo and D’Annibale 2014). In Fig. 3b the contour lines $\mu =\mathrm {const}$, are displayed (gray curves in the figure); each contour lines divides the plane in a stable region (marked with *S*) and in an unstable one (marked with *U*). There exists a contour line, i.e. a value of $\mu$, namely $\mu =0.33=:\mu _{d}^{min}$, below which the column is stable for any $\xi _{1}, \xi _{2}$. The figure shows, from a quantitative point of view, the consequences of the paradox discussed qualitatively above, namely that damping has a detrimental effect in the $\left( \xi _{1},\xi _{2}\right)$-plane, since, in except for a small region (filled in gray in the figure), which is close to an *optimal* damping ratio (dashed line in the figure), $\mu _{d}$ is lower with respect to $\mu _{c}$. Moreover, it can be shown that on the left side of the optimal direction the stability is governed by the first mode, while on the right side by the second one.

Previous results can be explained via asymptotic analysis, based on the evaluation of the eigenvalue sensitivities of the circulatory system when a small damping is introduced as a perturbation. Differently from the common approach (Kirillov 2005), and by rediscovering a pioneering idea contained in Andreichikov and Yudovich (1974), we suggested in Luongo and D’Annibale (2014, 2015) to start the asymptotic expansion not from the critically loaded circulatory system ($\mu =\mu _c$) but rather from a sub-critically loaded undamped system ($\mu <\mu _{c}$), with $\mu$ arbitrary in the interval $\left( \mu _{d}^{min},\mu _{c}\right)$ and taken constant; in this case a perturbation of a simple eigenvalue has to be carried out instead of a not-semi-simple (defective) double eigenvalue. The perturbation algorithm illustrated in Luongo and D’Annibale (2014, 2015) leads to the determination of the first eigenvalue sensitivities of the complex eigenvalues of the undamped column (see the “Appendix” for the details), namely:

7 $$\begin{aligned} {\hat{\uplambda }}_{j}\left( \mu \right) =-\frac{1}{2}{{\mathbf{v}}}_{j}^{T}\left( \mu \right) {\mathbf{C}}\mathbf{u}_{j}\,\left( \mu \right) \end{aligned}$$

being $\mathbf{u}_{j}$ and ${\mathbf{v}}_{j}$ the (real) right and left eigenvectors, respectively, and $j=1$ or 2. Therefore, the first sensitivity ${\hat{\uplambda }}_{j}$ is found to be real; the sign of $\underset{j}{\max }\left[ {\hat{\uplambda }}_{j}\left( \mu \right) \right]$ governs the stability of the system: if it is negative, the sub-critically loaded system remains stable, if it is positive, the damping renders the system unstable. By equating ${\hat{\uplambda }}_{j}=0$, see, e.g. Luongo and D’Annibale (2014), the asymptotic Whitney’s umbrella surface (labeled by *As*), showed in Fig. 3a, is obtained, together with the the contour lines $\mu =\mathrm {const}$ displayed in Fig. 3b (black lines). Remarkably, the asymptotic procedure furnishes an excellent representation of the exact surface, in particular, when the damping ratio produces high destabilizing effect; as it is expected, the approximation worsens close to the optimal direction, where an interaction between the eigenvalues occurs and the system becomes nearly-defective.

Finally, it is important to remark that the asymptotic procedure recalled above has not to be regarded as a mere perturbation algorithm, since it is able to explain the true essence of the paradox, that is: when a generic damping matrix is added to an undamped circulatory system in sub-critical regime, modes that would be marginally stable can become incipiently unstable. In this perspective, *no discontinuities appear in the damped system with respect to the undamped one;* thus, the apparent discontinuity of the amazing paradox is a consequence of a wrong point of view, in which the damped system is compared with the unique critically loaded undamped system, instead that with the infinitely many sub-critically loaded members of the undamped family.

### Post-critical behavior

The post-critical behavior of the Ziegler column is investigated in the following with the aim to analyze the amplitude of the limit-cycle when the load is close to the critical load of the damped system $\mu {}_{d}$. To this end, we will refer to two damped systems, marked with a black dot and a label I or II in Fig. 3b, respectively far and close enough to $\mu _{c}$, namely:

- case study I: $\xi _{1}=0.016, \xi _{2}=0.1$, entailing $\mu _{d}\simeq 0.65$, for which damping has a *strong destabilizing* effect (−69 %);
- case study II: $\xi _{1}=0.081, \xi _{2}=0.06$, entailing $\mu _{d}\simeq 1.63$ for which damping has a *moderate destabilizing* effect (−22 %).

**Fig. 4:**
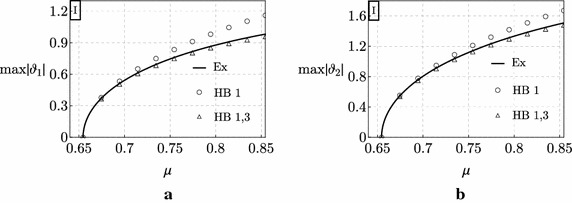
Bifurcation diagrams of the Ziegler column for the case study I: **a** $\max \left| \vartheta _{1}\right|$ versus $\mu$; **b** $\max \left| \vartheta _{2}\right|$ versus $\mu$

**Fig. 5:**
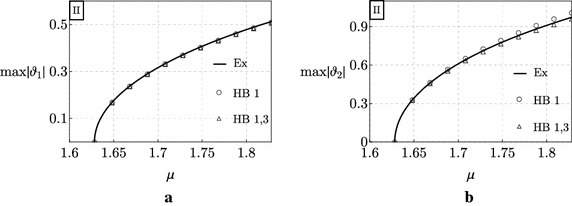
Bifurcation diagrams of the Ziegler column for the case study II: **a** $\max \left| \vartheta _{1}\right|$ versus $\mu$; **b** $\max \left| \vartheta _{2}\right|$ versus $\mu$

In Figs. 4 and 5 the exact bifurcation diagrams (black curves labeled with Ex) for the uncontrolled Ziegler column are displayed for the case study I and II, respectively. They have been obtained numerically via a continuation algorithm directly applied to the system (1). Figures 4 and 5 show the maximum values of the moduli of the amplitudes of the motion components, $\max \left| \vartheta _{1}\right|$ and $\max \left| \vartheta _{2}\right|$, which are plotted *vs* the bifurcation parameter $\mu$.

It is observed that, even when the bifurcation parameter slightly exceeds the critical value $\mu _{d}$, the column manifests large amplitude limit-cycles. This is due to destabilizing effect of damping which persists also in the post-critical regime. As a matter of fact, if we consider, for example, an increment of the load with respect to the critical value, $\delta \mu :=\mu -\mu _{d}$ equal to $\delta \mu = 0.1,$ we found: $\max \left| \vartheta _{1}\right| \simeq 0.75$ rad, $\max \left| \vartheta _{2}\right| \simeq 1.12$ rad in case I, and $\max \left| \vartheta _{1}\right| \simeq 0.37$ rad, $\max \left| \vartheta _{2}\right| \simeq 0.72$ rad in case II. Remarkably, we can conclude from this example that, for the same increment of the load, *the higher the destabilizing effect on linear stability, the higher the amplitude of the limit-cycle occurring in the post-critical regime*.

Finally, the same Figs. 4 and 5 show the results furnished by the Harmonic Balance Method applied to the two case studies. It is observed that, when the first harmonic is considered (points represented by small circles in the figures), the approximation of the exact results is good only in the case study II, while it worsens when the destabilizing effect of damping is significant (case study I). When instead the first and the third harmonics are considered (points represented by small triangles in the figures) the approximation of the exact results is excellent in both the case studies.

## The Nicolai paradox

**Fig. 6:**
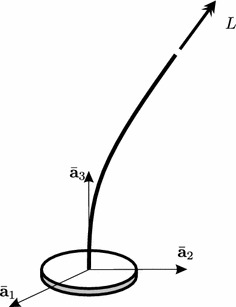
The Nicolai beam

The Nicolai beam, displayed in Fig. 6, is an elastic cantilever beam embedded in a 3D Euclidean space, of length $\ell$ and mass per unit length $\tilde{m}$, loaded at the tip by a follower torque of intensity *L*. In the present paper we will consider a discretized model of this beam, which has been obtained by using the Galerkin Method, with the aim to discuss the effects of a small vanishing torque on the linear and nonlinear stability. In particular, we will discuss some interesting results of the linear stability analysis, thus giving an explanation of the paradox, and we will introduce some new aspects occurring in the post-critical behavior. Here, damping has not been accounted for.

Following the notation of the Eq. (1), the linear operator and the trilinear forms associated to the nonlinearities of the discretized system, in nondimensional form, read:

8 $$\begin{aligned} \begin{aligned} {\mathbf{x}}&:=\left[ \begin{array}{c} x_{1}\\ x_{2} \end{array}\right] \\ {\mathbf{M}}&:=\left[ \begin{array}{cc} m &{} 0\\ 0 &{} m \end{array}\right] ,\quad {\mathbf{K}}:=\left[ \begin{array}{cc} J_{2}\omega ^{2} &{} 0\\ 0 &{} J_{1}\omega ^{2} \end{array}\right] \\ {\mathbf{C}}&:=\mathbf{0},\quad {\mathbf{H}}:=\left[ \begin{array}{cc} 0 &{} \, c_{\mu }\\ -\, c_{\mu } &{} 0 \end{array}\right] \\ {\mathbf{F}}_{1}\left( {\mathbf{x}},{\mathbf{x}},{\mathbf{x}}\right)&:=\left[ \begin{array}{c} \mu \, d_{1}\left( x_{2}x_{1}^{2}+x_{2}^{3}\right) +J_{2}d_{2}x_{1}^{3}+\left( J_{1}d_{3}+J_{2}d_{4}\right) x_{1}x_{2}^{2}\\ -\mu \, d_{1}\left( x_{1}x_{2}^{2}+x_{1}^{3}\right) +J_{1}d_{2}x_{2}^{3}+\left( J_{1}d_{4}+J_{2}d_{3}\right) x_{2}x_{1}^{2} \end{array}\right] \\ {\mathbf{F}}_{2}\left( {\mathbf{x}},{\dot{\mathbf{x}}},{\dot{\mathbf{x}}}\right)&:=\left[ \begin{array}{c} m\, d_{5}\left( x_{1}{{\dot{x}}}_{1}^{2}+x_{1}{\dot{x}}_{2}^{2}\right) \\ m\, d_{5}\left( x_{2}{\dot{x}}_{2}^{2}+x_{2}{\dot{x}}_{1}^{2}\right) \end{array}\right] \\ {\mathbf{F}}_{3}\left( {\mathbf{x}},{\mathbf{x}},{\ddot{\mathbf{x}}}\right)&:=\left[ \begin{array}{c} m\, d_{6}\left( x_{1}^{2}{{\ddot{x}}}_{1}+x_{1}x_{2}{\ddot{x}}_{2}\right) \\ m\, d_{6}\left( x_{2}^{2}{\ddot{x}}_{2}+x_{2}x_{1}{\ddot{x}}_{1}\right) \end{array}\right] \end{aligned} \end{aligned}$$

in which $x_{1}\left( t\right) , x_{2}\left( t\right)$ are (time-dependent) amplitudes of the trial function adopted for the Galerkin projection, $m, J_{1},J_{2}$ and $\mu$ are the nondimensional mass, inertia moments with respect to the two principal inertia axes and intensity of the follower torque, respectively, defined as:

9 $$\begin{aligned} \begin{aligned}m&=\frac{\tilde{m}}{m_{0}},&J_{1}&=\frac{\tilde{J_{1}}}{J_{0}},&J_{2}&=\frac{\tilde{J_{2}}}{J_{0}},&\mu&=\frac{L\ell }{EJ_{0}}\end{aligned} \end{aligned}$$

where *E* is Young modulus of the elastic material, $m_{0}$ and $J_{0}$ are the mass per unit length and the inertia moment, respectively, taken as the characteristics of an ideal symmetric system, from which the actual system can be generated via a perturbation (Luongo et al. 2014). The other quantities appearing in Eq. (8) are numerical coefficients resulting from the Galerkin projection, which has been performed by selecting as trial function, in each of the principal inertia plane, the first eigenfunction of the cantilever beam; they assume the following values:

10 $$\begin{aligned} \begin{aligned}\omega&=3.52,&c_{\mu }&=-3.79,&d_{1}&=7.18,&d_{2}&=-13.81\\ d_{3}&=6.41,&d_{4}&=-20.22,&d_{5}&=-4.60,&d_{6}&=-2.44 \end{aligned} \end{aligned}$$

It is important to remark that the continuous model from which the discretized equations of motion have been derived, has been formulated by modeling the Nicolai beam as a one-dimensional polar continuum, geometrically nonlinear and internally constrained. In particular, the constraints are the unshearability, the inextensibility and the untwistability. The first two are commonly used in the modeling of beams while the third one is based on an analysis of the orders of magnitude of the energy contribution of the underlying elastic model, according to Luongo and Zulli (2013). Once the kinematics is established, the partial integro-differential equations of motion are derived with the methods presented in Paolone et al. (2006) and Luongo and Zulli (2013) and expanded up to cubic terms.

### Linear analysis

By using the discretized system introduced above, we want to highlight the bifurcation mechanisms which guides the Nicolai paradox. When the follower torque $\mu$ is equal to zero (i.e. the system is Hamiltonian) and the two inertia moments are equal (i.e. $J_{1}=J_{2}=1$), the system admits a couple of purely imaginary coincident eigenvalues, see Fig. 1c; moreover, they are semi-simple, since two independent (real) eigenvectors, describing the same modal shape in the two planes, are associated with each of them. When the Hamiltonian system is loaded by small nonconservative forces, the two coalescent eigenvalues split on opposite parts of the complex plane (Seyranian and Mailybaev 2011; Seyranian et al. 2014; Luongo et al. 2014; Seyranian and Glavardanov 2014), see Fig. 1c, thus entailing instability; the presence of a small asymmetry, that we label with a parameter $\alpha$, is able to shift the critical load of a small amount only (Seyranian and Mailybaev 2011; Seyranian et al. 2014; Luongo et al. 2014; Seyranian and Glavardanov 2014).

Following the perturbation methods detailed in Seyranian and Mailybaev (2011), Seyranian et al. (2014), Luongo et al. (2014) and Seyranian and Glavardanov (2014), the splitting mechanism of the semi-simple eigenvalue $i \omega _j$ (here $j=1$), is described by the formula (see “Appendix” for details):

11 $$\begin{aligned} \uplambda ^{\pm }=i\omega _{j}+{\hat{\uplambda }}^{\pm } \end{aligned}$$

where ${\hat{\uplambda }}^{\pm }$ are the first sensitivities of the coalescent eigenvalues. Accordingly, the trivial equilibrium is asymptotically stable when $\mathrm {Re}\left( \uplambda ^{\pm }\right) <0$, i.e. when $\mathrm {Re}\left( {\hat{\uplambda }}^{\pm }\right) <0$, which leads to the following stability condition in the parameter space $\left( \alpha ,\mu \right)$:

12 $$\begin{aligned} \mu ^{2}\le c_{\alpha }^{2}\alpha ^{2} \end{aligned}$$

This latter equation represents the key point to understand the Nicolai paradox since, if the system is symmetric (i.e. $\alpha =0$), the critical value of the follower torque which produces dynamic instability is equal to zero. By referring to an elliptical cross section, for which the nondimensional inertia moments and the mass per unit length admit a series expansion in term of the splitting parameter $\alpha$ (see, e.g., Luongo et al. 2014 for further details):

13 $$\begin{aligned} \begin{aligned}m&=1+\alpha ,&J_{1}&=1+\alpha ,&J_{2}&=1+3\alpha +O\left( \alpha ^{2}\right) \end{aligned} \end{aligned}$$

the coefficient $c_{\alpha }$ in Eq. (12) assumes the value 3.26.

**Fig. 7:**
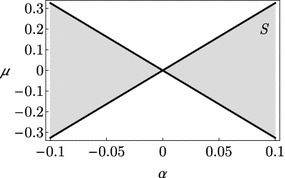
Stability domain in the $(\alpha ,\mu )$-plane. Stable zone *S* in *gray*

The stability domain expressed by Eq. (12) is displayed in Fig. 7. It is worth noticing that, since in the present paper a reduced two degrees of freedom system is considered, the effects of higher modes is ignored. However, the effects of this reduction needs to be deeply investigated since, e.g., when $\alpha =0$, all the modes are incipiently unstable, thus implying an infinite dimensional Center Manifold, which represents an open problem. In that sense the present paper is a first (although quite rough) approach to the matter.

### Post-critical behavior

**Fig. 8:**
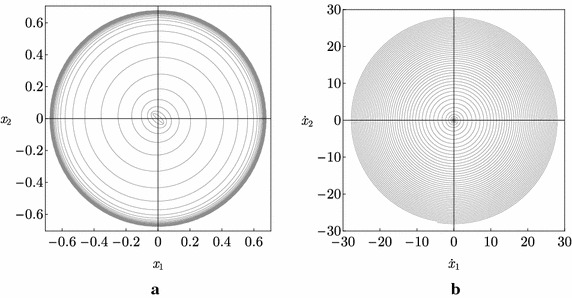
Numerical integration of the equations of motion ($\mu =0.5, \alpha =0, x_{1}\left(0 \right)=-x_{2}\left(0 \right)=0.03, {\dot{x}}_{1}\left(0 \right)={\dot{x}}_{2}\left(0 \right)=0$): **a** trajectory in the $(x_{1},x_{2})$-plane; **b** trajectory in the $({\dot{x}}_{1},{\dot{x}}_{2})$-plane

Our post-critical analysis is aimed to numerically investigate the dynamics of the *nonlinear* system close to the bifurcation point. To this end, a direct integration of the nonlinear equations of motion and a parametric analysis has been performed, in the case of elliptical cross section of the previous paragraph. Results of this integration are displayed in Fig. 8, from which the following considerations are drawn.

1. The system manifests a paradoxical behavior also in nonlinear regimes: indeed, starting with a couple of parameters $\left( \alpha ,\mu \right)$ that belongs to the unstable zone (the white one in Fig. 7), the system reaches, after a transient motion, a circular trajectory in the space of configuration variables, i.e. $\left( x_1\left( t \right) ,x_2\left( t \right) \right)$, whose amplitude is large and independent from the selected numerical parameters (see Fig. 8a); this circular motion is also unaffected by the choice of the initial conditions.
2. Once the large circular motion has been reached, the system increases its velocity unboundedly (see Fig. 8b). Therefore, the beam whirls with an increasing velocity, experiencing a conical motion.
3. The only effect played by the initial conditions and by the value of $\left( \alpha ,\mu \right)$ concerns the time requested by the system to reach the circular motion.

Finally, it is important to remark that, in the first approach to the nonlinear problem carried out in the present paper, the effects of higher modes are ignored since, as we said, a reduced model of two degrees of freedom has been considered. Moreover, damping has been ignored. The investigation of these aspects will be object of our studies in future works.

## The failure of the ‘similarity principle’

This section is devoted to detect the failure of the ‘similarity principle’ which occurs in linear and nonlinear behavior of PEM systems whose motion is described by equations in the form of Eq. (3). To this end, we will take as a prototype the piezoelectric-controlled Ziegler column (Ziegler 1952) depicted in Fig. 2b. The mechanical system is that analyzed in “The Ziegler paradox” section with the reference to the discussion of the Ziegler paradox. Moreover, it is equipped with two piezoelectric devices indicated with the symbols $\mathrm {Pz}_{1}$ and $\mathrm {Pz_{2}}$ in the figure, respectively: these latter are of capacitances $C_{1}^{P}:=2C_{P}, C_{2}^{P}:=C_{P}$, equal stiffnesses $k_{1}^{P}=k_{2}^{P}:=k_{P}$ (in the following we will considered this stiffness negligible with respect to the stiffness of the springs), equal coupling coefficients $g_{1}^{P}=g_{2}^{P}:=g$. Piezoelectric devices are placed in the correspondence of the ground and of the intermediate hinges, respectively, and each of them is connected to a joint of a two-nodes *active* circuit (sketched in the Fig. 2b), of equal inductances $L_{1}=L_{2}:=L$ and resistances $R_{1}$ and $R_{2}$, respectively, and to the ground.

The equations of motion of the PEM system are expressed in terms of its Lagrangian coordinates, namely the rotations of the two bars viz. $\vartheta _{1}$ and $\vartheta _{2}$, (see Fig. 2c) and the flux linkages at the circuit nodes $\psi _{1}$ and $\psi _{2}$, respectively. When an expansion up to the cubic terms in displacements and velocities of the exact kinematics of the mechanical system is developed, the ‘similarity principle’ is enforced and the piezoelectric mechanical stiffness is neglected [see D’Annibale et al. (2015) for more details on the derivation process], the equations of motion assume the form of Eq. (3), where definitions of Eq. (4) hold and:

14 $$\begin{aligned} \begin{aligned} {\mathbf{y}}&:=\left[ \begin{array}{c} \psi _{1}\\ \psi _{2} \end{array}\right] \\ {\mathbf{G}}&:=\left[ \begin{array}{cc} 1 &{} 0\\ -1 &{} 1 \end{array}\right] \end{aligned} \end{aligned}$$

Moreover, the quantities defined in (5), together with (tilde removed):

15 $$\begin{aligned} \tilde{\mathbf{y}}=\frac{1}{\psi _{0}}{\mathbf{y}},\quad \gamma =\frac{g}{\omega \ell \sqrt{mC_{P}}},\quad \psi _{0}=\ell \sqrt{\frac{m}{C_{P}}} \end{aligned}$$

are used for nondimensionalization (accounting for $C_{1}^{P}=2C_{P}, C_{2}^{P}=C_{P}, L_{1}=L_{2}=L, g_{1}^{P}=g_{2}^{P}=g$) and $\psi _{0}$ is a scaling flux-linkage.

### Linear analysis

Let us first consider the linearized equations (3), with the aim to discuss the bifurcation mechanism occurring in this paradox. First, we neglect the electro-mechanical coupling, by letting $\gamma =0$. The similarity principle then entails that the mechanical (primary) and electrical (secondary) sub-systems possess the same spectrum of the eigenvalues: if an eigenvalue is simple for the primary (or the secondary) sub-system taken alone, it is semi-simple for the whole PEM system. When the load $\mu$ reaches a critical value $\mu _{d}$, that is the smallest $\mu$ at which a mechanical (or electrical) eigenvalue (together with its complex conjugate) crosses from the left the imaginary axis (see Fig. 1b), a *simple Hopf bifurcation* occurs for the primary (or the secondary) sub-system.

When the electro-mechanical coupling is accounted for, i.e. $\gamma >0$, and the load is kept fixed at $\mu =\mu _{d}$, the bifurcation mechanism occurring in the PEM system is analogous to that of Nicolai, namely, the semi-simple eigenvalues split on the opposite part of the complex plane, thus entailing instability (Fig. 1c). What it is surprising in this paradox is that a vanishingly small gyroscopic coupling, which is introduced with the aim to control the mechanical system, i.e. to increase its critical load, produces instead instability (D’Annibale et al. 2015). Said in other words, the splitting, which represents the most valuable beneficial effect brought by an added device in the classical Den Hartog oscillator under external excitation, is, indeed, cause of instability in the autonomous nonconservative case.

**Fig. 9:**
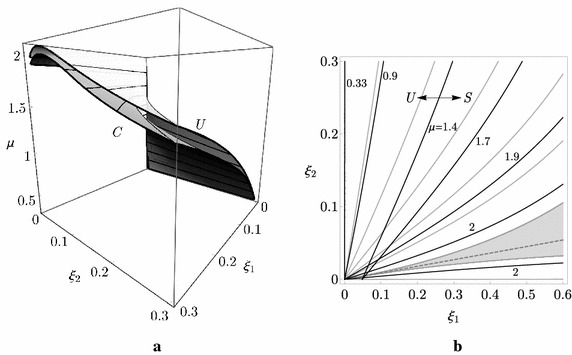
Controlled Ziegler column when $\gamma =0.05$: **a** critical manifold in the $(\mu ,\xi _{1},\xi _{2})$-parameter space: *C* controlled system, *U* uncontrolled system; **b** $\mu$-isolines: *S* stable region, *U* unstable region, *black curves* controlled system, *gray curves* uncontrolled system

The linear stability analysis of the PEM system can be carried out through an exact or an asymptotic analysis. Concerning the exact analysis, the stability domain of the controlled case can be obtained by using the Routh–Hurwitz criterion on the characteristic equation of the algebraic eigenvalue problem associated with the linearized Eq. (3). In the present paper, however, we built-up the stability domain numerically, i.e. by evaluating the eigenvalues, for a fixed $\gamma$, and in each point of a discretized portion of the $\left( \mu ,\xi _{1},\xi _{2}\right)$-space. Results relevant to the linear bifurcation scenario of the controlled system, when $\gamma =0.05$, are displayed in Fig. 9. In particular, Fig. 9a shows a comparison, in the $\left( \mu ,\xi _{1},\xi _{2}\right)$-space, of the critical surfaces of the controlled (marked with a *C* in the figure) and uncontrolled (marked with a *U* in the figure) systems, respectively. It is evident that the effect of the controller is in decreasing the stable region in the whole space considered (the surface of the controlled system is below that of the uncontrolled one). This effect is much more evident when the contour lines, displayed in Fig. 9b, are considered: indeed, the curves corresponding to the controlled system (in solid black) are on the right side (i.e. in the stable region) of those corresponding to the uncontrolled system (in solid gray), thus entailing an extension of the unstable region.

It is possible to show the detrimental effect of the gyroscopic coupling also via a perturbation method, by following the lines of D’Annibale et al. (2015), in which the first sensitivity of the semi-simple eigenvalue of a general continuous PEM system is determined, when a small coupling acts as a perturbation. By keeping fixed the load at $\mu =\mu _{d},$ the semi-simple eigenvalue $\uplambda _{0}$ at bifurcation splits according to Eq. (11), namely $\uplambda ^{\pm }=\uplambda _{0}\pm i\gamma \sqrt{b_{1}b_{2}}$, where $b_1,\,b_2$ are coefficients depending on the right and left eigenvectors of the uncontrolled sub-systems and on the gyroscopic matrix (see “Appendix” for details). It is apparent that (a) if the product $b_{1}b_{2}$ is complex or real and negative, one of the two roots has positive real part, thus entailing instability; (b) if the product $b_{1}b_{2}$ is real and positive, the two roots are purely imaginary, so that $\gamma$ is neutral at the first order. It is concluded that* a similar controller has a detrimental (or at most neutral) effect on stability.*

### Post-critical behavior

Preliminary results concerning the post-critical behavior of the controlled Ziegler column are discussed in this section, with the aim to investigate the effects of the controller on the large amplitude limit-cycles occurring in the uncontrolled case (see “Post-critical behavior” section referred to the Ziegler paradox). To this end, we directly integrated the nonlinear equations of motion (3) in the case studies I and II (marked in Fig. 3b), i.e. we selected the same two damped systems discussed with the reference to the post-critical behavior of the uncontrolled Ziegler column.

**Fig. 10:**
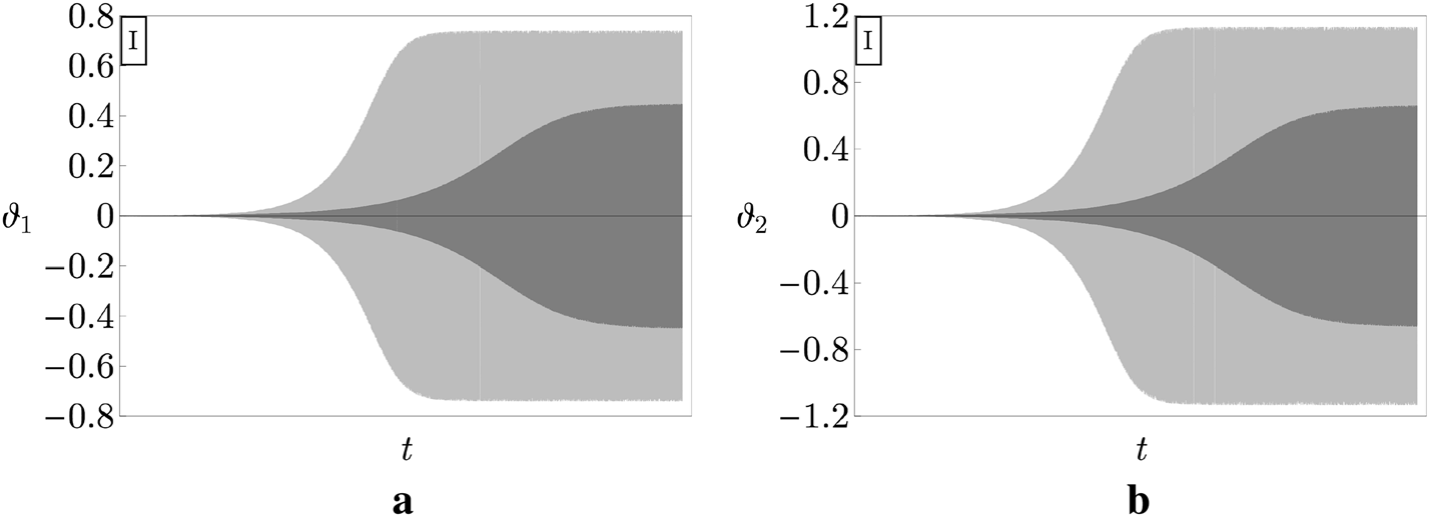
Time histories relevant to the case study I for the uncontrolled (*dark gray curves*) and controlled (*light gray curves*), when $\mu =0.68$ and $\gamma =0.05$: **a** $\vartheta _{1}$ versus *t*; **b** $\vartheta _{2}$ versus *t*

A comparison between the time histories of the components of motion $\vartheta _{1}$ and $\vartheta _{2}$, in uncontrolled and controlled ($\gamma =0.05$) systems, relevant to the case study I, when $\mu =0.68$, is presented in Fig. 10. It is seen that, for small increments of the load with respect the critical value, i.e. $\delta \mu =0.03$ in the uncontrolled case and $\delta \mu =0.05$ in the controlled one, the limit-cycle of the PEM system is stable, even if its amplitude (displayed in light gray in Fig. 10) is larger than the amplitude of the uncontrolled column (displayed in dark gray in the figure). Thus, *the similar controller increases the amplitude of the limit-cycle of the uncontrolled Ziegler column*, causing a detrimental effect.

**Fig. 11:**
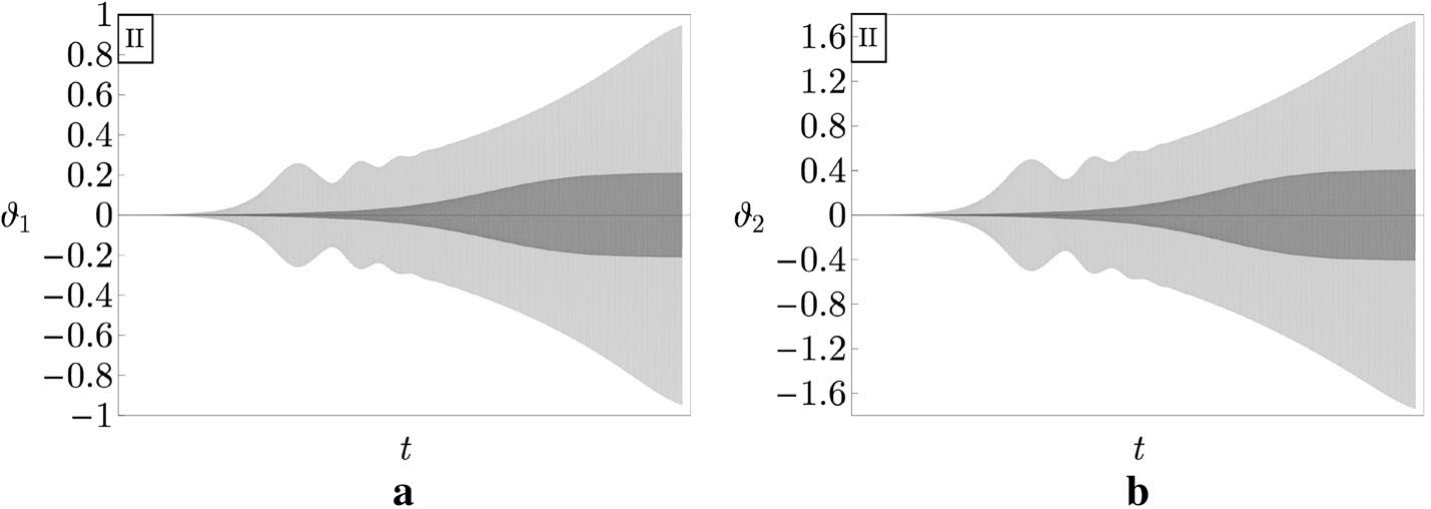
Time histories relevant to the case study II for the uncontrolled (*dark gray curves*) and controlled (*light gray curves*), when $\mu =1.66$ and $\gamma =0.01$: **a** $\vartheta _{1}$ versus *t*; **b** $\vartheta _{2}$ versus *t*

An even more dangerous situation is illustrated in Fig. 11, in which the time histories of $\vartheta _{1}$ and $\vartheta _{2}$, in uncontrolled and controlled $(\gamma =0.01)$ systems, relevant to the case study II, when $\mu =1.66$, are plotted. Indeed, for small increments of the load with respect the critical one (of the same order of the previous case, i.e. $\delta \mu =0.03$ in the uncontrolled system and $\delta \mu =0.1$ for the controlled one), the PEM system is unstable and the time histories of $\vartheta _{1}$ and $\vartheta _{2}$ diverge in time. Accordingly, the numerical integration has been truncated in the figures, just before this event. In contrast, the uncontrolled column experiences a stable limit-cycle. Thus, in this case, *the similar controller has a catastrophic effect in the post-critical regime of the controlled Ziegler column.*

The two examples shown are peculiar of two different behaviors met in numerically analyzing the nonlinear dynamics of a number of systems. Namely, when a system is chosen, and the load increased beyond the critical value, the behavior of Fig. 10 is initially found, then the behavior of Fig. 11 manifests itself when the load exceeds a threshold value, this latter depending on the system characteristics. In conclusion, the detrimental effect of the gyroscopic coupling in similar PEM systems persists also in the nonlinear regime. This aspect of the problem needs a more deep investigation, which will be object of forthcoming papers.

## Conclusions

In this paper, some amazing paradoxical phenomena, well- and less-known in the literature, concerning linear dynamic stability of mechanical systems, have been studied referring to finite-dimensional prototype systems. Paradoxes concern: (a) the destabilizing effect of damping, or Ziegler paradox; (b) the zero critical value of the load, or Nicolai paradox; (c) the failure of the similarity principle in controlling stability by piezoelectric devices. For all these problems, an explanation has been given, based on asymptotic expansions of the eigenvalues, started by simple or double and semi-simple eigenvalues. In the Ziegler case, a procedure different from that usually adopted in literature (starting from a double and not-semi-simple eigenvalue) has been followed, able to reveal the true essence of the paradox.

Some new results, concerning investigations on the nonlinear behavior of the three prototype systems, have been provided, although the analysis has been so far of purely numerical type. The following conclusions have been drawn.

1. The Ziegler column experiences stable large-amplitude limit-cycles. The more destabilizing the damping, the larger the amplitude of the limit-cycle. Therefore, the loss of stability (in the Lyapunov sense), is not a mere mathematical aspect of the problem, but a signal of an incoming dangerous phenomenon from an engineering point of view (more interested in the amplitude of the oscillations than in the quality of the equilibrium).
2. The Nicolai beam also suffers large-amplitude circular motion in the space of configuration variables, even when a very small follower torque is applied, and, quite surprisingly, irrespectively of the chosen parameters and initial conditions. Even worse, this motion occurs at increasing velocity, diverging to infinite, representing a new paradoxical phenomenon existing in the nonlinear field. This unrealistic result is conjectured to depend on the absence of damping, so far not included in the model. Further investigations are therefore needed also considering the effects of damping, higher modes and twistability.
3. The PEM Ziegler column, possesses double eigenvalues when a ‘similar’ control system is adopted, requiring an active circuit. This equipment, that previous studies have shown to be optimal in controlling external excitations, is instead detrimental in controlling stability. When the motion is analyzed in the post-critical range, both stable and unstable large-amplitudes limit-cycles exist, the former close to bifurcation, the latter far from bifurcation. In both cases, however, the oscillations of the controlled system are larger than those of the uncontrolled system, so that the similar control is detrimental even in the nonlinear field.

The previous results, to be corroborated by analytical studies, denote that the paradoxes produce their malefic effects also in the nonlinear range.

## Appendix: Asymptotic analysis

This Appendix is devoted to furnish details about asymptotic analyses specifically developed for the investigation of the three paradoxes. The algorithms are here discussed with the aim to render the paper self-contained; they have been already presented in Luongo and D’Annibale (2015), Luongo and D’Annibale (2014) and Andreichikov and Yudovich (1974), with reference to the Ziegler paradox, in Seyranian and Mailybaev (2011), Seyranian et al. (2014), Luongo et al. (2014) and Seyranian and Glavardanov (2014), for the Nicolai paradox, and in D’Annibale et al. (2015) for the Failure of the ‘similarity principle’.

### Ziegler paradox

The linear part of the equations of motion (1) reads:

16 $$\begin{aligned} {\mathbf{M}}{\ddot{\mathbf{x}}}+{\mathbf{C}}{\dot{\mathbf{x}}}+\left( {\mathbf{K}}+\mu {\mathbf{H}}\right) {\mathbf{x}}=\mathbf{0} \end{aligned}$$

By letting ${\mathbf{x}}=\mathbf{w}\exp \left( \uplambda t\right)$ the following eigenvalue problem is obtained:

17 $$\begin{aligned} \left[ \uplambda ^{2}{\mathbf{M}}+\uplambda {\mathbf{C}}+\left( {\mathbf{K}}+\mu {\mathbf{H}}\right) \right] \mathbf{w}=\mathbf{0} \end{aligned}$$

being $\left( \uplambda ,\mathbf{w}\right)$ the eigenpairs of the system.

We aim to tackle the problem (17) by a perturbation method (see, e.g., Luongo and D’Annibale 2014). To this end we first rescale the damping as ${\mathbf{C}}\rightarrow \varepsilon {\mathbf{C}}$, where $0<\varepsilon \ll 1$ is a perturbation parameter artificially introduced and to be reabsorbed at the end of the procedure. Then, we perform the series expansions:

18 $$\begin{aligned} \begin{aligned} \uplambda&=\uplambda _{0}+\varepsilon {\hat{\uplambda }}+\cdots \\ \mathbf{w}&=\mathbf{w}_{0}+\varepsilon \hat{\mathbf{w}}+\cdots \end{aligned} \end{aligned}$$

in which the coefficients ${\hat{\uplambda }}, \hat{\mathbf{w}}$, are unknowns and proportional to the relevant $\varepsilon$-derivatives evaluated at $\varepsilon =0$. By substituting Eq. (18) in the problem (17) and requiring that this latter must be satisfied for any $\varepsilon$, the following perturbation equations are obtained:

19 $$\begin{aligned} \begin{aligned}\varepsilon ^{0}:&\left[ \uplambda _{0}^{2}{\mathbf{M}}+\left( {\mathbf{K}}+\mu {\mathbf{H}}\right) \right] \mathbf{w}_{0}=\mathbf{0}\\ \varepsilon ^{1}:&\left[ \uplambda _{0}^{2}{\mathbf{M}}+\left( {\mathbf{K}}+\mu {\mathbf{H}}\right) \right] \hat{\mathbf{w}}=-\uplambda _{0}\left( {\mathbf{C}}+2{\hat{\uplambda }}{\mathbf{M}}\right) \mathbf{w}_{0} \end{aligned} \end{aligned}$$

with $\mu$ (here having the meaning of follower force parameter) arbitrary in the interval $\left( \mu _{d}^{min},\mu _{c}\right)$. The generating Eq. (19-a) admits the solution $\left( \uplambda _{0},\mathbf{w}_{0}\right) =\left( \pm i\omega _{j},\mathbf{u}_{j}\right)$, i.e. the eigenpairs of the sub-critically loaded undamped system, with $\mathbf{u}_{j}$ real. Since, however, the problem is not self-adjoint for the presence of the nonconservative force, the right eigenvectors are *not* mutually orthogonal, so that the left (real) eigenvectors ${\mathbf{v}}_{j}$, which form the dual basis, are also of interest. Right and left eigenvectors satisfy, respectively:

20 $$\begin{aligned} \begin{aligned}&\left[ \left( {\mathbf{K}}+\mu {\mathbf{H}}\right) -\omega _{j}^{2}{\mathbf{M}}\right] \mathbf{u}_{j}=\mathbf{0}\\&\left[ \left( {\mathbf{K}}+\mu {\mathbf{H}}^{T}\right) -\omega _{j}^{2}{\mathbf{M}}\right] {\mathbf{v}}_{j}=\mathbf{0} \end{aligned} \end{aligned}$$

Moreover, they are bi-orthogonal with respect to the mass matrix, according to ${\mathbf{v}}_{k}^{T}{\mathbf{M}}\mathbf{u}_{j}=0$ if $k\ne j$, and they are normalized to satisfy ${\mathbf{v}}_{j}^{T}{\mathbf{M}}\mathbf{u}_{j}=1$. To evaluate the sensitivity of $i\omega _{j}$ to the damping, we take $\uplambda _{0}=i\omega _{j},\,\mathbf{w}_{0}=\mathbf{u}_{j}$ in Eq. (19-a) and substitute them in Eq. (19-b); then the solvability condition of this latter, i.e. the known term must be orthogonal to the kernel of the adjoint operator, furnishes the Eq. (7) ($\varepsilon$ reabsorbed).

### Nicolai paradox

The linearized equations of motion of the undamped Nicolai discretized beam, i.e. when ${\mathbf{C}}=\mathbf{0}$, are formally equivalent to Eq. (16). The system depends on two parameters, namely $\mu$ (here having the meaning of follower torque parameter) and $\alpha$, accounting for the small asymmetries of the mass and stiffness. In particular, the matrices ${\mathbf{M}}$ and ${\mathbf{K}}$ in Eq. (16) admit the following Mc Laurin series expansion:

21 $$\begin{aligned} \begin{aligned} {\mathbf{M}}&={\mathbf{M}}_{0}+\alpha {\mathbf{M}}_{\alpha }+O\left( \alpha ^{2}\right) \\ {\mathbf{K}}&={\mathbf{K}}_{0}+\alpha {\mathbf{K}}_{\alpha }+O\left( \alpha ^{2}\right) \end{aligned} \end{aligned}$$

where ${\mathbf{M}}_{0},{\mathbf{K}}_{0}$ are symmetric and positive-definite matrices of the ideal symmetric mechanical system.

The eigenvalue problem (17) is solved by a perturbation method (see, e.g., Luongo et al. 2014). When $\alpha =\mu =0$, due to the symmetry of the system, the eigenvalue $\uplambda {}_{0}$ is a double semi-simple eigenvalue. In such case expansions given by Eq. (18) hold (Seyranian and Mailybaev 2003) and, moreover, the following parameter rescaling is introduced:

22 $$\begin{aligned} \begin{aligned} \left( \alpha ,\mu \right) \rightarrow&\varepsilon \left( \alpha ,\mu \right) \end{aligned} \end{aligned}$$

By using Eqs. (18), (21) and (22) in Eq. (17), the following perturbation equations are obtained:

23 $$\begin{aligned} \begin{aligned}\varepsilon ^{0}:&\left[ \uplambda_{0}^{2}{\mathbf{M}}_{0}+{\mathbf{K}}_{0}\right] \mathbf{w}_{0}=\varvec{0}\\ \varepsilon ^{1}:&\left[ \uplambda _{0}^{2}{\mathbf{M}}_{0}+{\mathbf{K}}_{0}\right] \hat{\mathbf{w}}=-\left[ 2\uplambda _{0}{\hat{\uplambda }}{\mathbf{M}}_{0}+\alpha \left( \uplambda _{0}^{2}{\mathbf{M}}_{\alpha }+{\mathbf{K}}_{\alpha }\right) +\mu {\mathbf{H}}\right] \mathbf{w}_{0} \end{aligned} \end{aligned}$$

The generating solution is $\left( \uplambda _{0},\mathbf{w}_{0}\right) =\left( \pm i\omega _{j},\mathbf{U}\mathbf{a}\right)$, where $j=1,2,\ldots n_{m}/2$ (being $n_{m}$ odd). Moreover, $\mathbf{a}:=\left( a_{1},a_{2}\right) ^{T}$ is a column-vector listing two unknown amplitudes $a_{1}$ and $a_{2}, \mathbf{U}:=\left( \mathbf{u}_{1},\mathbf{u}_{2}\right)$ is the $n_{m}\times 2$ modal matrix listing the real right eigenvectors $\mathbf{u}_{1}$ and $\mathbf{u}_{2}$ associated with $\uplambda _{0}$, which are mutually orthogonal and normalized according to $$\begin{aligned}\mathbf{u}_{i}^{T}{\mathbf{M}}_{0}\mathbf{u}_{j}=\delta _{ij}\end{aligned}$$. We take $\uplambda _{0}= i \omega _j$ and by using these results, the Eq. (23-b) reads:

24 $$\begin{aligned} \left[ \uplambda _{0}^{2}{\mathbf{M}}_{0}+{\mathbf{K}}_{0}\right] \hat{\mathbf{w}}=-\left[ 2\uplambda _{0}{\hat{\uplambda }}{\mathbf{M}}_{0}+\alpha \left( \uplambda _{0}^{2}{\mathbf{M}}_{\alpha }+{\mathbf{K}}_{\alpha }\right) +\mu {\mathbf{H}}\right] \mathbf{U}\mathbf{a} \end{aligned}$$

Solvability condition of this latter furnishes:

25 $$\begin{aligned} \left[ 2\uplambda _{0}{\hat{\uplambda }}\mathbf{I}+\alpha \left( \uplambda _{0}^{2}\hat{\mathbf{M}}_{\alpha }+\hat{{\mathbf{K}}}_{\alpha }\right) +\mu \hat{{\mathbf{H}}}\right] \mathbf{a}=\mathbf{0} \end{aligned}$$

where:

26 $$\begin{aligned} \hat{\mathbf{M}}_{\alpha }:=\mathbf{U}^{T}{\mathbf{M}}_{\alpha }\mathbf{U},\qquad \hat{{\mathbf{K}}}_{\alpha }:=\mathbf{U}^{T}{\mathbf{K}}_{\alpha }\mathbf{U},\qquad \hat{{\mathbf{H}}}:=\mathbf{U}^{T}{\mathbf{H}}\mathbf{U} \end{aligned}$$

are $2\times 2$ matrices and $\mathbf{I}$ is the $2\times 2$ identity matrix; moreover, $\mathbf{U}^{T}{\mathbf{M}}_{0}\mathbf{U}=\mathbf{I}$ has been used and it should be remarked that, in this case, left eigenvectors coincide with the right ones. Equation (25) is an eigenvalue problem in the unknown ${\hat{\uplambda }}$. Its solutions can be determined by solving the following characteristic equation:

27 $$\begin{aligned} {\hat{\uplambda }}^{2}+\mathcal {I}_{1}{\hat{\uplambda }}+\mathcal {I}_{2}=0 \end{aligned}$$

where:

28 $$\begin{aligned} \mathcal {I}_{1}:=\frac{1}{2\uplambda _{0}}\mathrm {tr}\left[ \alpha \left( \uplambda _{0}^{2}\hat{\mathbf{M}}_{\alpha }+\hat{{\mathbf{K}}}_{\alpha }\right) +\mu \hat{{\mathbf{H}}}\right] ,\quad \mathcal {I}_{2}:=\frac{1}{4\uplambda _{0}^{2}}\mathrm {det}\left[ \alpha \left( \uplambda _{0}^{2}\hat{\mathbf{M}}_{\alpha }+\hat{{\mathbf{K}}}_{\alpha }\right) +\mu \hat{\mathbf{H}}\right] \end{aligned}$$

are linear and quadratic (complex) invariants, respectively. Eq. (27) admits, in general, two distinct roots ${\hat{\uplambda }}^{\pm }$; therefore, the splitting of the double semi-simple eigenvalue $i\omega _{j}$ is furnished by Eq. (11) ($\varepsilon$ reabsorbed).

### Failure of the ‘similarity principle’

The linear part of the equations of motion (3) reads:

29 $$\begin{aligned} \begin{aligned}{\left\{ \begin{array}{ll} {\mathbf{M}}{\ddot{\mathbf{x}}}+{\mathbf{C}}{\dot{\mathbf{x}}}+\left( {\mathbf{K}}+\mu {\mathbf{H}}\right) {\mathbf{x}}-\gamma {\mathbf{G}}^{T}{\dot{\mathbf{y}}}=\mathbf{0}\\ {\mathbf{M}}{\ddot{\mathbf{y}}}+{\mathbf{C}}{\dot{\mathbf{y}}}+\left( {\mathbf{K}}+\mu {\mathbf{H}}\right) {\mathbf{y}}+\gamma {\mathbf{G}}{\dot{\mathbf{x}}}=\mathbf{0} \end{array}\right. }\end{aligned} \end{aligned}$$

By letting ${\mathbf{x}}=\mathbf{w}\exp \left( \uplambda t\right) , {\mathbf{y}}=\mathbf{z}\exp \left( \uplambda t\right)$ the following eigenvalue problem is obtained:

30 $$\begin{aligned} {\left\{ \begin{array}{ll} \left[ \uplambda ^{2}{\mathbf{M}}+\uplambda {\mathbf{C}}+\left( {\mathbf{K}}+\mu {\mathbf{H}}\right) \right] \mathbf{w}-\gamma \uplambda {\mathbf{G}}^{T}\mathbf{z}=\mathbf{0}\\ \\ \left[ \uplambda ^{2}{\mathbf{M}}+\uplambda {\mathbf{C}}+\left( {\mathbf{K}}+\mu {\mathbf{H}}\right) \right] \mathbf{z}+\gamma \uplambda {\mathbf{G}}\mathbf{w}=\mathbf{0} \end{array}\right. } \end{aligned}$$

The eigenvalue problem (30) is solved, also in this case, by using a perturbation method. First we rescale $\gamma \rightarrow \varepsilon \gamma$ and we fix the load at $\mu =\mu _{d}$, where $\mu _{d}$ is the critical load value of the uncontrolled PEM system, for which a Hopf bifurcation occurs. Then, the series expansions (18) hold and, moreover:

31 $$\begin{aligned} \begin{aligned}&\mathbf{z}=\mathbf{z}_{0}+\varepsilon \hat{\mathbf{z}}+\cdots \end{aligned} \end{aligned}$$

Accordingly, the relevant perturbation equations read:

32 $$\begin{aligned} \begin{aligned}\varepsilon ^{0}:&{\left\{ \begin{array}{ll} \begin{aligned}\left[ \uplambda _{0}^{2}{\mathbf{M}}+\uplambda _{0}{\mathbf{C}}+\left( {\mathbf{K}}+\mu _{d}{\mathbf{H}}\right) \right] \mathbf{w}_{0}=\mathbf{0}\\ \\ \left[ \uplambda _{0}^{2}{\mathbf{M}}+\uplambda _{0}{\mathbf{C}}+\left( {\mathbf{K}}+\mu _{d}{\mathbf{H}}\right) \right] \mathbf{z}_{0}=\mathbf{0} \end{aligned} \end{array}\right. }\\ \\ \varepsilon ^{1}:&{\left\{ \begin{array}{ll} \begin{aligned}\left[ \uplambda _{0}^{2}{\mathbf{M}}+\uplambda _{0}{\mathbf{C}}+\left( {\mathbf{K}}+\mu _{d}{\mathbf{H}}\right) \right] \hat{\mathbf{w}}= &{} -{\hat{\uplambda }}\left( 2\uplambda _{0}{\mathbf{M}}+{\mathbf{C}}\right) \mathbf{w}_{0}+\gamma \uplambda _{0}{\mathbf{G}}^{T}\mathbf{z}_{0}\\ \\ \left[ \uplambda _{0}^{2}{\mathbf{M}}+\uplambda _{0}{\mathbf{C}}+\left( {\mathbf{K}}+\mu _{d}{\mathbf{H}}\right) \right] \hat{\mathbf{z}}= &{} -{\hat{\uplambda }}\left( 2\uplambda _{0}{\mathbf{M}}+{\mathbf{C}}\right) \mathbf{z}_{0}-\gamma \uplambda _{0}{\mathbf{G}}\mathbf{w}_{0} \end{aligned} \end{array}\right. } \end{aligned} \end{aligned}$$

The $\varepsilon ^{0}$-order problem is a linear eigenvalue problem in which the electrical sub-system doubles the uncontrolled mechanical one and the (double) semi-simple eigenvalues are $\uplambda _{0}=\uplambda _{0j}\,,j=1,2,\ldots ,n_{m}$. However, since $\mu =\mu _{d}$, there exists a (here assumed unique) *j* for which $\mathrm {Re}\left( \uplambda _{0j}\right) =0$ and $\mathrm {\frac{\partial }{\partial \mu }Re}\left( \uplambda _{0j}\right) _{\mu _{d}}>0$, so that a Hopf bifurcation occurs, when $\mathrm {Im}\left( \uplambda _{0j}\right) =:\pm \omega _{j}\ne 0$. Therefore, we take $\uplambda _{0}=i\omega _{j}$ and, accordingly, the generating solution of Eq. (32-a) reads:

33 $$\begin{aligned} \left( \begin{array}{c} \mathbf{w}_{0}\\ \mathbf{z}_{0} \end{array}\right) =a_{1}\left( \begin{array}{c} \mathbf{u}_{j}\\ \mathbf{0} \end{array}\right) +a_{2}\left( \begin{array}{c} \mathbf{0}\\ \mathbf{u}_{j} \end{array}\right) \end{aligned}$$

where $a_{1},\,a_{2}$ are arbitrary constants and $\mathbf{u}_{j}$, is the (right and complex) *j*-th eigenvector of the uncontrolled system associated with $\uplambda _{0}$. In order to evaluate the sensitivity of $\uplambda _{0}$ to the coupling parameter $\gamma$, we substitute the previous findings in Eq. (32-b), thus obtaining:

34 $$\begin{aligned} \begin{aligned}\varepsilon ^{1}:&{\left\{ \begin{array}{ll} \begin{aligned}\left[ \uplambda _{0}^{2}{\mathbf{M}}+\uplambda _{0}{\mathbf{C}}+\left( {\mathbf{K}}+\mu _{d}{\mathbf{H}}\right) \right] \hat{\mathbf{w}}= &{} -{\hat{\uplambda }}\left( 2\uplambda _{0}{\mathbf{M}}+{\mathbf{C}}\right) a_{1}\mathbf{u}_{j}+\gamma \uplambda _{0}{\mathbf{G}}^{T}a_{2}\mathbf{u}_{j}\\ \\ \left[ \uplambda _{0}^{2}{\mathbf{M}}+\uplambda _{0}{\mathbf{C}}+\left( {\mathbf{K}}+\mu _{d}{\mathbf{H}}\right) \right] \hat{\mathbf{z}}= &{} -{\hat{\uplambda }}\left( 2\uplambda _{0}{\mathbf{M}}+{\mathbf{C}}\right) a_{2}\mathbf{u}_{j}-\gamma \uplambda _{0}{\mathbf{G}}a_{1}\mathbf{u}_{j} \end{aligned} \end{array}\right. }\end{aligned} \end{aligned}$$

Solvability condition of Eq. (34) leads to a characteristic equation in the unknown ${\hat{\uplambda }}$ in the form of Eq. (27), where $\mathcal {I}_{1}=0, \mathcal {I}_{2}=\gamma ^{2}b_{1}b_{2}$ and the following definitions hold:

35 $$\begin{aligned} \begin{aligned}b_{1}:=&\uplambda _{0}{\mathbf{v}}_{j}^{H}{\mathbf{G}}^{T}\mathbf{u}_{j},\qquad b_{2}:=\uplambda _{0}{\mathbf{v}}_{j}^{H}{\mathbf{G}}\mathbf{u}_{j}\end{aligned} \end{aligned}$$

Moreover, the superscript *H* denotes the complex-conjugate and ${\mathbf{v}}_{j}$ is the (left and complex) *j*-th eigenvector of the uncontrolled system, solution of the following problem:

36 $$\begin{aligned} \left[ \bar{\uplambda }_{0}^{2}{\mathbf{M}}+\bar{\uplambda }_{0}{\mathbf{C}}+\left( {\mathbf{K}}+\mu _{d}{\mathbf{H}}\right) \right] {\mathbf{v}}_{j}=\mathbf{0} \end{aligned}$$

Equation (27) admits two (generally) distinct roots ${\hat{\uplambda }}^{\pm }$ and, therefore, the splitting of the double semi-simple eigenvalue $i\omega _{j}$ is given by Eq. (11) ($\varepsilon$ reabsorbed).
